# Improving coordination, proprioception, balance and motor proficiency in Down syndrome with developmental games

**DOI:** 10.1113/EP092739

**Published:** 2025-05-22

**Authors:** Alireza Rezaee, Hasan Daneshmandi, Hesam Ramezanzade, Sahar Mohammadzadeh, Mert Kurnaz, Mustafa Altınkök

**Affiliations:** ^1^ Sport Injuries and Corrective Movements Department Gilan University Rasht Iran; ^2^ Department of Sport Sciences, School of Humanities Damghan University Damghan Iran; ^3^ Department of Physical Education and Sport Teaching, Faculty of Sport Sciences Haliç University Istanbul Türkiye; ^4^ Department of Physical Education and Sport Teaching, Faculty of Sport Sciences Akdeniz University Antalya Türkiye

**Keywords:** corrective games, Down syndrome, gross and fine motor proficiency, Oseretsky test, sense of position

## Abstract

This study aimed to investigate the impact of corrective‐developmental games on proprioception, coordination, balance and motor proficiency in individuals with Down syndrome. The current quasi‐experimental study with pre‐test and post‐test design explores the impact of corrective‐developmental games (fine‐gross motor skill games and univariate‐multivariate games) on the proprioception, coordination, balance and motor proficiency of individuals with Down syndrome. The research sample comprises 50 individuals with Down syndrome, with an average age of 17.38 years, divided into two groups: intervention (13 men and 13 women) and control (12 men and 12 women). After the pre‐test, the intervention group participated in a series of games, including fine‐gross games and univariate–multivariate games, for 8 weeks (24 1‐h sessions in total). Mid‐test and post‐test were conducted after 4 and 8 weeks, respectively. To assess balance, the Stork test (test–retest reliability: 0.59), Sharpened Romberg test (reliability: 0.76–0.91) and Y Balance test (reliability: 0.84–0.87) were used. The Purdue Pegboard test and knee position sense with a goniometer (open and closed eyes) evaluated eye–hand coordination and sense of position. The Bruininks–Oseretsky test (retest coefficient: 0.78–0.86) measured motor proficiency. Repeated measures ANOVA revealed significant improvements in the intervention group for static balance (Stork test, *P *= 0.001), dynamic balance (Y test, *P *= 0.001), position sense (open eyes: *P *= 0.001, closed eyes: *P *= 0.001), two‐hand coordination (*P *= 0.001), preferred hand coordination (*P *= 0.001), gross motor proficiency (*P *= 0.001), upper limb coordination (*P *= 0.001) and total motor proficiency (*P *= 0.001). The intervention group significantly outperformed the control group post‐intervention on all measures except the Stork test and fine motor proficiency. The findings of this research confirm the beneficial effect of games‐based interventions on motor fitness and corrective‐developmental indicators in Down syndrome. Future studies should investigate the long‐term impact on daily life activities and generalizability to similar populations. The results have potential implications for designing effective interventions to enhance motor skills in individuals with Down syndrome.

## Abstract (highlights)

- **What is the central question of this study?**
  What is the impact of corrective‐developmental games on proprioception, coordination, balance and motor proficiency in individuals with Down syndrome?
- **What is the main finding and its importance?**
  There are beneficial effect of games‐based interventions on motor fitness and corrective‐developmental indicators in Down syndrome. The results have potential implications for designing effective interventions to enhance motor skills in individuals with Down syndrome.

## 1 INTRODUCTION

Down syndrome, also known as trisomy 21, is a genetic disorder caused by the presence of an extra chromosome 21 (Esbensen et al., 2024). This extra genetic material causes a range of physical and cognitive symptoms, including distinctive facial features, intellectual disability, and an increased risk for certain medical conditions including orthopaedic, cardiovascular, musculoskeletal and perceptual impairments (Akhtar & Bokhari, 2020). Historically, medical and educational interventions for individuals with Down syndrome focused primarily on managing the physical health complications associated with the condition. In recent decades, there has been an increased emphasis on early intervention programmes, special education, and therapies to support the cognitive, social and emotional development of individuals with Down syndrome and maximize their potential (Suarez‐Villadat et al., 2025).

Individuals with Down syndrome often experience cognitive and motor impairments. According to a study by Grieco et al. (2015), these individuals exhibit significant limitations in cognitive, adaptive functioning (which refers to an individual's ability to use their cognitive skills to adapt and function independently in daily life), which affects a person's ability to learn, think and problem‐solve. (Grieco et al., 2015). People with Down syndrome typically have an IQ in the range of 50–70, which is considered a mild to moderate intellectual disability (Abd El‐Hady et al., 2018).

In addition to cognitive impairment, people with Down syndrome may also experience considerable difficulties with motor functions. These include initiating and maintaining movements, reduced balance, scarce muscle tone, limited voluntary control and coordination difficulties, delayed motor development, and gross and fine motor skills (Al‐Nemr & Reffat, 2024; Jain et al., 2021). These motor problems can affect a person's ability to participate in activities of daily life, such as clothing, grooming, and self‐care, as well as more complex movements like walking, running, participating in physical activities and playing sports (Alesi et al., 2018).

Motor skill development in individuals with Down syndrome is often delayed, impacting their ability to perform tasks that require physical coordination and control (Wentz et al., 2021). They may also have visual and auditory impairments that can affect their ability to process sensory information necessary for maintaining balance (Valencia‐Jiménez et al., 2023). Postural control and balance can be a challenge for individuals with Down syndrome due to muscle weakness, low muscle tone and altered body proportions (Pelosi et al., 2020). However, there are several methods, such as physical therapy, occupational therapy, assistive gadgets, yoga and sport, that can help improve postural control (Moriello et al., 2020).

Individuals with Down syndrome may experience balance problems due to delayed cerebellar maturation and smaller cerebellum and brainstem, which are critical for postural control and balance. Proprioception refers to the body's ability to sense its position, movement, and forces in relation to its surroundings; it is often reduced in these individuals, contributing to motor deficits and joint instability (Valencia‐Jiménez et al., 2023).

Individuals with Down syndrome exhibit a range of physical and cognitive symptoms, among which hypotonia is notable. Hypotonia, or low muscle tone, is a condition where muscles are less firm than usual, and it is a common feature in people with Down syndrome. This condition contributes to delayed motor development, poor posture, and difficulties with balance and coordination (Galli et al., 2014; Valencia‐Jiménez et al., 2023). The underlying causes of hypotonia in Down syndrome include abnormal nervous system development and reduced muscle fibre density. Physical therapy and exercise programmes are essential in managing hypotonia and enhancing motor skills in these individuals (Valencia‐Jimenez et al., 2020).

Physical therapy and other interventions can be effective in improving postural control, balance, coordination and overall physical function in individuals with Down syndrome. Exercises that target the cerebellum and other areas of the brain involved in balance can help improve coordination and stability. Balance training, gait training and other interventions can also be effective in improving postural control and reducing the risk of falls and injury (Memon et al., 2024).

In this research, we examined the role of developmental and corrective games. Games that can be challenging, involving fantasy and curiosity, can be controlled. If the game surprises the player with unfulfilled expectations through various activities, it will be a pleasure (Macedo et al., 2015). Developmental and corrective games are designed to help individuals improve specific skills and abilities through fun and engaging activities (Syahputri & Sukoco, 2020). These games can be particularly helpful for individuals with developmental or physical challenges, such as those with Down syndrome. Our study is based on the BCP learning package, a developmental and corrective game structure that aims to improve gross motor skills, balance, coordination and proprioception skills for people with Down syndrome.

## 2 METHODS

The current research employs a quasi‐experimental design with a pre‐test–post‐test methodology. Data for the study were collected through fieldwork, and the samples were deliberately selected using a non‐randomized approach. As a result, this research can be categorized as applied research. The Ethics Committee of Sports Sciences Research Institute (SSRI) (Protocol ID: IR.SSRC.REC.1399.018) approved all protocols employed in this study. The principles outlined in the Declaration of Helsinki were meticulously adhered to throughout the entirety of this research endeavor.

### 2.1 Participants

A cohort of 50 adolescents with Down syndrome, comprising both girls and boys, was drawn from the government vocational training centre. The center offers free courses, certification exams, and training programs, focusing on both general workforce development and specialized programs for disadvantaged groups, including people with disabilities. The participants' average age was 17.3 years (SD 1.8), with all individuals being above the age of puberty (14–25 years). The sample was divided into two groups: an intervention group consisting of 26 participants (comprising 13 girls and 13 boys), and a control group comprising 24 participants (equally split between girls and boys). While the intervention group discontinued their involvement in the government vocational training centre sports programme, the control group adhered to the government vocational training centre sports programme, engaging in 1‐h sessions under the guidance of a sports coach, three times a week. These activities were varied in different sessions, but the majority of the institute's activities included light warm‐up (walking, running, static stretching movements, rotational movement, dynamic stretching movements), axial activities (including flexion and extension of the trunk, rotation around the longitudinal axis of the body, limited movements in different directions), running in different directions, jumping over obstacles, throwing a ball towards each other, shooting the ball towards each other and towards the goal, etc. The inclusion criteria encompassed individuals aged 14 to 25 years, possessing an IQ ranging from 50 to 70 (considered educable), and in good physical health (absence of bone fracture or strain or muscle tear, absence of diseases that reduce the energy required to perform). Additionally, none of the participants had previously engaged in balance exercises. Exclusion criteria involved the presence of cardiovascular ailments or the manifestation of symptoms thereof, as well as any concurrent neurological conditions during the protocol's execution. Individuals taking specific medications (e.g., seizure control drugs, behavioural disorder medications) during the research implementation, those expressing dissatisfaction or lacking cooperation, and those unwilling to sustain their participation in the assessments were also excluded.

### 2.2 Measurement

In this study, we employed valid and reliable tests to measure and quantify the research variables, which included parameters such as balance, coordination, sense of position and motor proficiency among individuals with Down syndrome. The following list of specific tests is used.

### 2.3 Balance assessments

#### 2.3.1 The Stork test

The Stork test is a commonly utilized method for assessing balance in individuals with Down syndrome. During this assessment, the subject stands in a unipedal position on a flat surface during the assessment, elevating the opposing leg to knee level while keeping both arms alongside the body (Ribeiro et al., 2021). The arms can move in any direction. A skilled examiner uses a timer to record the maximum duration during which the individual sustains this posture, and the measurement is complete when the participant places the unsupported foot back on the ground. This procedure is repeated twice for each leg, and the most favourable time is documented as the recorded outcome. The Stork test primarily assesses static balance. Notably, this test demonstrates robust intra‐examiner reliability (*r* = 0.87) while exhibiting varying degrees of retest reliability, ranging from poor to good (*r* = 0.59). Additionally, research suggests that test–retest reliability for the duration of one‐leg standing is consistently moderate among both children and adults.

#### 2.3.2 The Sharpened Romberg test

The Sharpened Romberg test entails placing the participant without shoes in a stance where one leg, known as the dominant leg, is placed in front of the other while crossing the arms over the chest. The time that an individual can maintain this position, both with open eyes and closed eyes, is recorded as their performance score (Lanska & Goetz, 2000). This test exhibits a strong level of reliability, ranging from 0.90 to 0.91 when administered with open eyes and from 0.76 to 0.77 when conducted with closed eyes (Steffen & Seney, 2008).

#### 2.3.3 The Y Balance test

The Y test is a valid and reliable test used to measure dynamic balance. Before initiating the test, participants' dominant leg was determined (Chimera et al., 2015). If the right leg was identified as dominant, the test was carried out in a counterclockwise direction; if the left leg was dominant, it was executed in a clockwise direction. Participants adopted a stance with their dominant leg at the centre‐line and performed a reaching movement with the opposite leg, followed by returning to the initial position using both legs.

Throughout the test, participants stretched their toes as far as possible within the specified directions. The distance from the point of contact to the centre‐line was noted as the reach distance, measured in centimetres. Each participant completed three trials for each direction, and the results were recorded. A 15‐s break was given after each attempt. Subsequently, the average reach distance was calculated for each direction. This average was then divided by the length of the dominant leg and multiplied by 100 to determine the reach distance as a percentage of leg length. The reliability of the Y test has been reported within a range of 0.84 to 0.087 (Shaffer et al., 2013).

### 2.4 Measurement of eye–hand coordination

#### 2.4.1 The Purdue Pegboard test

The Purdue Pegboard Model 32020 test is a comprehensive method for evaluating fingertip dexterity and eye‐hand coordination (Tiffin & Asher, 1948). This assessment comprises five distinct stages. The stages of the test are detailed as follows:

1. Dominant hand manipulation. During the first stage, participants are instructed to systematically extract pins one by one using their dominant hand. These pins are then accurately placed into matching holes aligned along the axis of the dominant side.
2. Non‐dominant hand manipulation. In the second stage, the focus shifts to assessing the proficiency of the non‐dominant hand. Participants are required to extract a pin from the top and securely position it into the embedded holes along the axis of the non‐dominant side.
3. Bilateral coordination. The third stage introduces a simultaneous manipulation challenge. Participants are expected to remove pins using both their dominant and non‐dominant hands, placing them accurately into embedded holes along two separate axes.
4. Composite score of initial stages. The fourth stage involves a composite score that is computed by adding together the performance scores achieved in the first three stages. This stage is not associated with a distinct physical task; rather, it combines the scores obtained from the preceding stages.
5. Assembly task. The final stage involves a more complex activity. Participants use both hands to perform a series of actions with a pin and washers. Initially, they use their dominant hand to insert a pin into a specified hole then using their non‐dominant hand, they add a washer onto the pin. Subsequently, they use their dominant hand again to stack another washer onto the one already placed. This process is repeated, where a washer is placed on top of the previously stacked one using the non‐dominant hand.

This test generates five distinct scores, each corresponding to the five stages. The allotted time varies across stages, with the first three stages being restricted to 30 s each, while the assembly stage extends to 60 s. This test has acceptable validity and reliability (Buddenberg & Davis, 2000).

### 2.5 Measuring the sense of the position

#### 2.5.1 Evaluation of knee position sense with a goniometer

Measuring knee position sense using a goniometer is a valid and reliable method (Irving et al., 2016). During the assessment procedure, participants were instructed to sit on a chair with the backrest inclined at an 80 degree angle relative to the horizon. To optimize alignment for accurate measurements, a specialized pad was strategically positioned beneath both thighs, near the knee joint. This adjustment ensured the leg's orientation perpendicular to the ground, creating a precise 90 degree angle with the thigh. To execute the evaluation, the goniometer was employed, with its fixed arm aligned to the femur and its movable arm aligned to the tibia. This set‐up was established to replicate the anatomical axis of rotation. To acquaint participants with the assessment protocol, a preliminary phase involved conducting the test with open eyes at various angles, repeated two to three times. Subsequently, a target angle of 60 degrees for knee flexion was designated. Participants were required to maintain this angle for 5 s while keeping their eyes closed. Following this, the leg was returned to the initial position of 90 degrees knee flexion. Participants were then instructed to reproduce the target angle by performing knee extension movements, both with eyes open and with eyes closed. Each participant underwent three attempts for both open‐ and closed‐eye conditions. The mean absolute error in replicating the target angle was computed across these attempts and recorded as the participant's final score.

### 2.6 Assessing motor proficiency

#### 2.6.1 The Bruininks–Oseretsky test

To evaluate the motor proficiency of the participants in this study, the complete version of the Bruininks–Oseretsky test was employed (Bruininks & Bruininks, 2005). This comprehensive assessment comprises a total of 46 items, distributed across eight sub‐tests. Among these, three sub‐tests encompass measures of reaction time, visual‐motor control, upper limb agility and fine motor performance. An additional set of four sub‐tests encompasses evaluations of running speed and agility, balance, bilateral coordination and strength, which collectively measure gross motor performance and provide a comprehensive assessment of the child's motor development status. Furthermore, one of the subtests is specifically designed to measure upper limb coordination. The combined scores from all eight sub‐tests play a crucial role in determining the overall general motor proficiency. It is worth highlighting that this assessment tool exhibits exceptionally high validity and has effectively proven its ability to differentiate between children with movement disorders and those who typically develop without such issues. Regarding its reliability, the retest coefficient for this assessment is 78% for the long form and 86% for the short form. Additionally, the inter‐rater reliability for the complete version falls within the range of 79–97%. Further psychometric analyses, involving various types of validity and reliability assessments, confirm the suitability and effectiveness of this instrument.

#### 2.6.2 Gaming protocol

When participants with Down syndrome arrived at the sports facility, the coaches were given the game package, marking the start of an 8‐week research intervention that included three 1‐h sessions every week. After a 12‐min warm‐up session, the subjects were grouped and given specific game programmes to follow at designated stations. At regular intervals, rotations were carried out, requiring the groups to move to the next stations. In this research, the games utilized for the study were classified based on the complexity of their execution, specifically distinguishing between single‐variable and multi‐variable games. Single‐variable games are games that focus on only one physical‐motor factor (balance or strength or …), while multi‐variable games involve several physical‐motor factors simultaneously (for example, the development of balance, gross motor function and hand–eye coordination). Both types of games (single‐variable and multi‐variable) were used in all sessions. Additionally, these games were divided into two distinct categories: individual and group, depending on the level of participant engagement. Some games focused exclusively on improving isolated variables, like balance, while others aimed to enhance multiple factors simultaneously, such as both balance and gross motor function. In contrast to earlier studies, both individual and group games were carefully designed to align movement goals with developmental improvements. It is crucial to emphasize that each game incorporated into the sessions was developed with the principle of overload in mind, effectively minimizing the risk of potential injuries during the training. The games were taught to the participants by trained instructors. The method of teaching each game, the number of repetitions, and the duration of each game session were specified. The instructors supervised the participants in the correct execution of the games. Table 1 presents a comprehensive overview of specific games showcased during the gaming sessions, offering details on game characteristics, objectives and the frequency of sessions.

**TABLE 1:** Games chosen according to content and objectives.

Task	Content	Objectives	Number of sessions
Several games were developed to enhance the balance of individuals with Down syndrome, both individually and within groups. One such game is referred to as the spiral game	Coach lays a suitable hemp rope flat on the ground. Players stand on the rope's edge and rotate as per the coach's command, gradually forming a spiral with the rope	Improving static and dynamic balance Enhancing gross motor function	1st, 4th, 7th, 9th, 13th, 18th, and 22nd sessions (total: seven sessions)
Several games were developed with the primary aim of enhancing both individual and group‐based hand–eye coordination of participants. A specific illustration of such games is the flying ball targeting game	Players are presented with multiple balls. Assistant coaches throw the balls into the air, and players are required to aim and target them	Enhancing hand–eye coordination. Improving fine motor function. Enhancing gross motor function	2nd, 5th, 8th, 12th, 15th, 19th and 23rd sessions (total: seven sessions)
A series of games were developed to cater to both individual and group settings, with a primary focus on augmenting the position sense of individuals with Down syndrome. One notable exemplar from this game series was the ‘Colourful Ball Tickle’ game	Participants would lie adjacent to a wall, placing the soles of their feet against it. Coloured balls were positioned beneath their feet. Participants were instructed to manipulate the balls using their feet, following specific directional cues	Amplifying position sense in the feet and knees of participants	1st, 4th, 7th, 9th, 13th, 18th, and 22nd sessions (total: seven sessions)
Some games (individually and in groups) were designed to enhance fine motor function in individuals with Down syndrome. An example is the pyramid puzzle game	Participants sit on the floor with an arrangement of glasses before them. Adjacent to them, on the coach's table, a stack of glasses is artfully configured into a pyramid shape. The objective involves aligning the glasses in front of the players to mirror the pyramid on the coach's table, using two small sticks held in their hands	Refinement of fine motor skills Enhancement of hand–eye coordination	4th, 7th, 9th, 13th, 18th, 21st and 24th sessions (total: seven sessions)
A series of games were devised with both individual and group formats, specifically aimed at enhancing the gross motor skills of individuals with Down syndrome. One such game is the Escape Balloons activity	The Escape Balloons game introduces an exhilarating experience where participants engage in capturing balloons in a designated hall setting. The game begins with the coach placing a bag of balloons just behind the starting line, strategically positioned in front of an air fan within the hall. Players enthusiastically pursue and capture the balloons as they are propelled by fan's airflow	Advancing gross motor skills. Enhancing dynamic balance. Improving hand–eye coordination	3rd, 5th, 8th, 12th, 15th, 18th, 21st and 24th sessions (total: eight sessions)
A variety of games were developed for both individual and group settings, with the goal of addressing all research variables. An example is the Porcelain Ball in a Porcelain Dish game	In the Porcelain Ball in a Porcelain Dish game, players engage in tossing coloured balls into designated coloured boxes on the ground. These boxes are arranged in multiple rows. The unique aspect involves players standing on a sponge shoe under one foot while slightly elevating the other foot from the ground. Subsequently, players execute ball throws using the hand	Enhancement of dynamic balance Refinement of hand–eye coordination Improvement in knee and sole position sense Advancement of fine motor skills Advancement of gross motor skills	3rd, 5th, 8th, 11th, 14th, 16th, 18th, 21st and 24th sessions (total: nine sessions)

The gaming equipment included a variety of components, coloured papers, coloured balls, balloons, balance boards, varying‐sized wooden elements, sponges, hemp rope, plastic chairs, oversized paper dice, plastic glasses, coloured cardboards, paper shooting targets, small coloured cards, lasers and an assortment of balls with diverse sizes (including bocce, goal ball, handball, futsal, soccer and volleyball balls, among others).

#### 2.6.3 Procedure

Before starting the game protocol, individuals with Down syndrome performed a pre‐test to evaluate variables including balance (the Stork test, the Sharpened Romberg test, and the Y Balance test), hand–eye coordination (the Purdue Pegboard test), sense of position (evaluation of knee position sense with goniometer) and motor proficiency (the Oseretsky test including three sub‐tests encompass measures of reaction time, visual‐motor control, upper limb agility, and fine motor performance. An additional set of four sub‐tests encompasses evaluations of running speed and agility, balance and bilateral coordination) was conducted. Each of the balance, hand–eye coordination, and position sense tests was performed three times, and the average of the three times was recorded. In the pre‐test, two familiarization trials were conducted before the three main trials. After 4 weeks, an intermediate assessment test was conducted, and subsequently, at the end of the eighth session, a final evaluation was carried out (both the intermediate and post‐test were administered similarly to the pre‐test). Additionally, prior to commencing the interventions, trainers and evaluators participated in multiple briefing and training sessions. These sessions facilitated their familiarity with the game content and equipped them with techniques for evaluating the research variables. Each session was 75 min long and included 8 min of preparation, 12 min of warm‐up, 45 min of play and 10 min of cool‐down. Games were played in each session with the aim of developing all dependent variables of the study, including static and dynamic balance, hand–eye coordination, sense of position, and gross and fine motor efficiency.

### 2.7 Data analysis

To facilitate the comparison of two groups at different test stages, we utilized repeated measures ANOVA. Before conducting this analysis, we investigate several assumptions. The Shapiro–Wilk test was employed to assess the normality of the data distribution. Also, we examined the homogeneity of variance within each of the two groups for every variable using Levene's test. We also assessed the homogeneity of variance–covariance across the test stages using Mauchly's test of sphericity (Supporting file). Additionally, to compare the two groups at specific test stages, we performed an independent Student's *t*‐test. All statistical analyses were executed using SPSS Statistics version 24 software (IBM Corp., Armonk, NY, USA), and the significance level was set at (*P *= 0.05).

## 3 RESULTS

This section explores an analysis of how the participants performed in terms of the research variables (balance, coordination, sense of position and motor development gain) during different test stages (pre‐test, mid‐test, post‐test) in both the intervention and the control groups. Table 2 shows the mean and standard deviation of the demographic characteristics of the research subjects.

**TABLE 2:** Demographic characteristics.

Characteristic	Male (*n* = 25)	Female (*n* = 25)	Total (*n* = 50)
Age (year)	17.59 (3.775)	17.10 (4.17)	17.4 (3.912)
Height (cm)	167.97 (6.355)	152.19 (7.988)	161.34 (19.538)
Weight (kg)	68.72 (6.187)	57.43 (5.473)	63.98 (8.113)

Data are means (SD).

**FIGURE 1:**
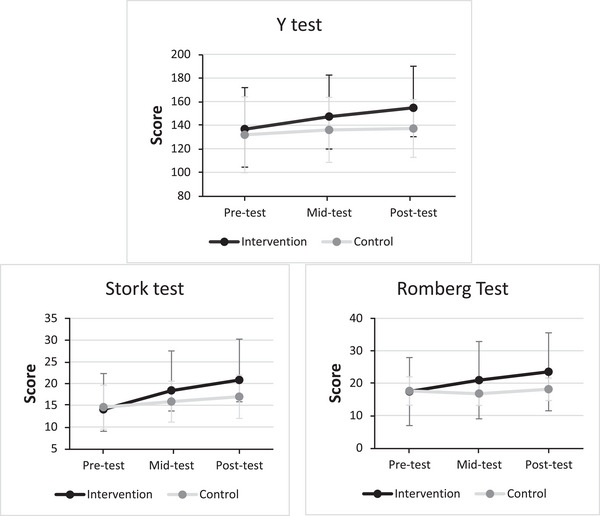
Average performance of subjects in balance tests across test stages (*n*_Intervention _= 26, *n*_Control _= 24).

We utilized repeated measures ANOVA to compare the participants’ performances. Before conducting the analysis, we ensured that standard assumptions were met and validated. These steps involved confirming whether the data distribution was normal within each group and test stage using the Shapiro–Wilk test. The results indicated that the data distribution for both experimental and control groups in the Y Balance test was normal across all three stages: pre‐test (*P*_E _= 0.7, *P*_C _= 0.22), mid‐test (*P*_E _= 0.65, *P*_C _= 0.38) and post‐test (*P*_E _= 0.69, *P*_C _= 0.36). The results demonstrated that the data distribution for the Stork Balance test was normal across pre‐test (*P*_E _= 0.08, *P*_C _= 0.118), mid‐test (*P*_E _= 0.061, *P*_C _= 0.923) and post‐test (*P*_E _= 0.082, *P*_C _= 0.738) phases, and similarly, the distribution for the Romberg Balance test exhibited normality throughout the pre‐test (*P*_E _= 0.092, *P*_C _= 0.916), mid‐test (*P*_E _= 0.067, *P*_C _= 0.507) and post‐test (*P*_E _= 0.153, *P*_C _= 0.495) stages. We also ensured that there was homogeneity of variance in the data between the experimental and control groups using Levine's test. The results indicated that homogeneity of variance was present between the experimental and control groups in the Y Balance test across all three stages: pre‐test (*P *= 0.262), mid‐test (*P *= 0.307) and post‐test (*P *= 0.287). Furthermore, homogeneity of variance was observed between the experimental and control groups for the Stork Balance test across the three stages of pre‐test (*P *= 0.876), mid‐test (*P *= 0.616) and post‐test (*P *= 0.952), as well as for the Romberg Balance test throughout the pre‐test (*P *= 0.464), mid‐test (*P *= 0.778) and post‐test (*P *= 0.672) phases. Additionally, we checked if the variance–covariance was consistent within the test stages using Mauchly's test of sphericity. Due to the violation of the assumption of homogeneity of variance–covariance in the Y Balance (*P *= 0.004) and Stork balance tests (*P *< 0.0001), the Greenhouse–Geisser statistic was reported. Table 3 displays the results of the repeated measures ANOVA, comparing the performance across the test stages in the Y semi‐dynamic balance test, Stork balance test, and Romberg balance test for both the experimental and control groups. Furthermore, Figure 1 visually illustrates the average scores from the balance tests, including the pre‐test, mid‐test and post‐test stages for both groups.

**TABLE 3:** Results of repeated measures ANOVA tests for comparing experimental and control groups in test stages for the balance tests.

Source	Sum of squares	df	*F*	*P*	Partial η^2^
Y test					
Test	3487.282	1.322	74.077	0.001	**0.6**07
Test × Intervention	1020.162	1.322	21.67	0.001	**0.3**11
Intervention	4681.075	1	1.538	0.221	**0.0**31
Error	2259.665	63.453			
Stork test					
Test	535.619	1.446	62.288	0.001	**0.55**6
Test × Intervention	124.632	1.446	14.494	0.001	**0.2**32
Intervention	144.684	1	0.954	0.334	**0.0**19
Error	412.754	69.403			
Romberg test					
Test	276.874	2	31.002	0.001	**0.3**92
Test × Intervention	211.584	2	23.699	0.001	**0.3**31
Intervention	366.626	1	1.672	0.202	**0.0**34
Error	428.536	96			

As illustrated in Figure 1, there are discernible patterns in the results of the Y, Romberg and Stork balance tests. The subjects in the experimental group exhibit more improvements in their performance.

Table 3 demonstrates that the interaction effect (test × intervention) is statistically significant across all three of the Y, Stork and Romberg tests. To further investigate the differences between the two groups at each stage of testing, we conducted independent *t*‐tests. Additionally, to examine within‐group comparisons across different testing stages, we employed a dependent *t*‐test. The results revealed noteworthy findings. In the Y tests (*t* = 2.026, *P *= 0.048, Cohen's *d* = 0.573) and Romberg tests (*t* = 2.183, *P *= 0.037, Cohen's *d* = 0.618), a significant difference between the experimental and control groups emerged during the post‐test stage. Conversely, no significant difference between the groups was observed in the Stork test (*t* = 1.823, *P *= 0.076, Cohen's *d* = 0.516). Furthermore, the outcomes of dependent *t*‐tests underscored substantial changes within the experimental group. Across all three of the Y, Stork and Romberg tests, the differences between the pre‐test, mid‐test and post‐test stages were statistically significant (*P* = 0.01). However, within the control group, significant discrepancies were limited to the Stork test between the pre‐test and post‐test stages, as well as in the Y test between the pre‐test and mid‐test stages (*P* = 0.05).

**FIGURE 2:**
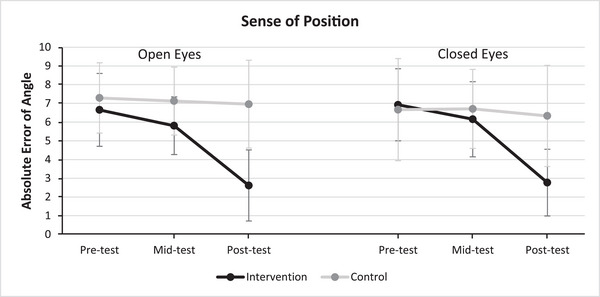
Average performance of participants in body position sense tests with both eyes open and eyes closed (*n*_Intervention _= 26, *n*_Control _= 24).

Figure 2 presents the mean scores of participants in the open‐eyes and closed‐eyes position sense tests across the pre‐test, mid‐test and post‐test stages for both the experimental and the control groups. It is evident that in both conditions of eyes open and eyes closed the intervention group consistently outperformed the control group in terms of position sense. Additionally, individuals in the intervention group significantly reduced their angular deviation from the pre‐test to the post‐test stage. Furthermore, a significant discrepancy was observed between the intervention and control groups during the post‐test phase.

To facilitate a comparison between the intervention and control groups across the test stages, repeated measures ANOVA was employed. The outcomes of this analysis are presented in Table 4. The results of the Shapiro–Wilk test indicated that the data distribution was normal in both open‐eye and closed‐eye conditions for the control group across all three stages of pre‐test (*P*_Open _= 0.126, *P*_Closed _= 0.06), mid‐test (*P*_Open _= 0.093, *P*_Closed _= 0.277) and post‐test (*P*_Open _= 0.2, *P*_Closed _= 0.71) and for the experimental group across all three stages of pre‐test (*P*_Open _= 0.068, *P*_Closed _= 0.661), mid‐test (*P*_Open _= 0.2, *P*_Closed _= 0.71) and post‐test (*P*_Open _= 0.154, *P*_Closed _= 0.188). Homogeneity of variance was observed between the experimental and control groups for open eyes across the three stages of pre‐test (*P *= 0.712), mid‐test (*P *= 0.303) and post‐test (*P *= 0.415), as well as for closed eyes throughout the pre‐test (*P *= 0.606), mid‐test (*P *= 0.853) and post‐test (*P *= 0.611) phases. Due to the violation of the assumption of homogeneity of variance–covariance in open eyes (*P *< 0.0001) and closed eyes (*P *< 0.0001), the Greenhouse–Geisser statistic is reported.

**TABLE 4:** Results of repeated measures ANOVA tests for comparing experimental and control groups in test stages for position sense.

Source	Sum of squares	df	*F*	*P*	Partial η^2^
Open eyes					
Test	63.312	1.064	4.94	0.029	**0.0**93
Test × Intervention	180.112	1.064	14.055	0.001	**0.22**6
Intervention	237.010	1	15.746	0.001	**0.2**47
Error	615.128	96			
Closed eyes					
Test	144.763	1.463	68.108	0.001	**0.5**87
Test × Intervention	101.136	1.463	47.583	0.001	**0.4**98
Intervention	62.052	1	4.732	0.035	**0.0**9
Error	102.024	96			

Table 4 displays that the interaction effect (test × intervention) attains statistical significance in both eyes‐open and eyes‐closed scenarios. Consequently, for scrutinizing the inter‐group disparities within each stage, an independent *t*‐test was implemented. Likewise, to examine intra‐group variations across test stages within both intervention and control groups, a dependent *t*‐test was applied. The findings underscore substantial insights. With eyes open, the intervention group exhibited significant superiority over the control group during the mid‐test (*t* = 2.758, *P *= 0.008, Cohen's *d* = 0.781) and post‐test (*t* = 4.273, *P *= 0.001, Cohen's *d* = 1.21). Moreover, in the context of eyes‐closed performance, a significant difference emerged between the two groups in the post‐test (*t* = 5.453, *P *= 0.001, Cohen's *d* = 1.544). Furthermore, outcomes from dependent *t*‐tests underscore the evolution within the intervention group. For both eyes‐open and eyes‐closed performance, significant differences between test stages were observed (*P *= 0.001). However, within the control group, significant variation was limited to the eyes‐closed condition, with a significant difference between pre‐test and post‐test stages (*P* = 0.01).

**FIGURE 3:**
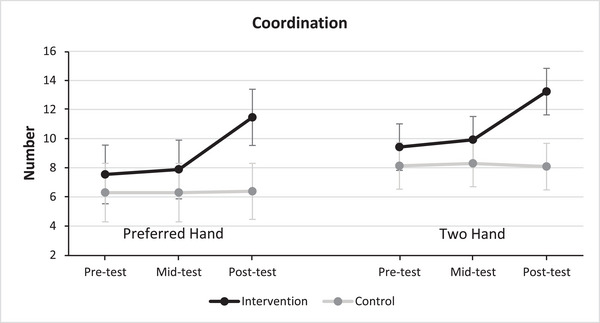
Mean scores of subjects in coordination tests for dominant and two‐handed conditions across test stages (*n*_Intervention _= 26, *n*_Control _= 24).

Figure 3 illustrates the average scores of participants in the coordination tests, specifically evaluating performance with the dominant hand and coordination involving both hands. The examination extends the pre‐test, inter‐test and post‐test phases for both the experimental and control groups. Notably, the intervention group demonstrated superior performance compared to the control group in both the dominant hand and two‐handed conditions, particularly evident during the post‐test stage.

Table 5 provides insights into the outcomes derived from repeated measures ANOVA. This analysis aimed to juxtapose the performance of the intervention and control groups across the various test stages. The results of the Shapiro–Wilk test indicated that the data distribution was normal in both preferred hand and two hand conditions for the control group across all three stages of pre‐test (*P*_PH _= 0.22, *P*_TH _= 0.06), mid‐test (*P*_Open _= 0.22, *P*_Closed _= 0.06) and post‐test (*P*_Open _= 0.29, *P*_Closed _= 0.06) and for the experimental group across all three stages of pre‐test (*P*_Open _= 0.29, *P*_Closed _= 0.316), mid‐test (*P*_Open _= 0.713, *P*_Closed _= 0.488) and post‐test (*P*_Open _= 0.839, *P*_Closed _= 0.646). Homogeneity of variance was observed between the experimental and control groups across the preferred hand condition in the three stages of pre‐test (*P *= 0.385), mid‐test (*P *= 0.141) and post‐test (*P *= 0.05), but not for the two hand condition throughout the pre‐test (*P *= 0.004), mid‐test (*P *= 0.003) and post‐test (*P *= 0.001) phases. (Although the assumption of homogeneity of variance was not met in the two‐hand condition, the repeated measures ANOVA test is robust against this assumption not being met.) Due to the violation of the assumption of homogeneity of variance–covariance in the open eyes (*P *< 0.0001) and closed eyes conditions (*P *< 0.0001), the Greenhouse–Geisser statistic is reported.

**TABLE 5:** Repeated measures ANOVA test results for comparing experimental and control groups in test stages for coordination.

Source	Sum of squares	df	*F*	*P*	Partial η^2^
Preferred hand					
Test	123.005	1.442	112.288	0.001	**0.7**01
Test × Intervention	112.605	1.442	102.795	0.001	**0.6**82
Intervention	261.356	1	15.289	0.001	**0.2**42
Error	52.581	96			
Two hand					
Test	100.811	1433	61.951	0.001	**0.5**63
Test × Intervention	113.531	1.433	69.768	0.001	**0.5**92
Intervention	824.345	1	38.337	0.001	**0.444**
Error	78.109	96			

Based on the results presented in Table 5, it appears that the interaction effects are statistically significant in both the dominant and two‐handed conditions. To better understand the differences between the groups that received the intervention and those in the control group at different stages of the test, we performed independent *t*‐tests. Similarly, comparing stages within each group, paired *t*‐tests were employed. The outcomes yielded noteworthy insights. A significant difference between the intervention and control groups was evident in both dominant (mid‐test, *t* = 2.275, *P *= 0.027, *d*_Cohen _= 0.644; post‐test, *t* = 6.949, *P *= 0.001, d_Cohen _= 1.967) and two‐handed conditions (mid‐test, *t* = 4.883, *P *= 0.001, d_Cohen _= 1.382; post‐test, *t* = 8.576, *P *= 0.001, d_Cohen _= 2.428). Furthermore, the outcomes of dependent *t*‐tests underscored significant disparities within the intervention group across all test stages encompassing both dominant and two‐handed conditions (*P *= 0.01). However, within the control group, no significant distinctions were observed across any of the test stages.

Finally, to compare the motor skills of the subjects, including gross motor skills, fine motor skills, upper limb coordination, and general motor skills, within the two intervention and control groups across the pre‐test and post‐test stages, repeated measures ANOVA were conducted. The data are presented in Table 6, illustrating the average performance and standard deviation of the subjects in motor skills and its subcategories across the intervention and control groups during the pre‐test and post‐test phases.

**TABLE 6:** Mean and standard deviation of motor proficiency scores (and subscales) in intervention and control groups across test stages.

	Gross motor proficiency		Fine motor proficiency		Upper‐limb coordination		Total motor proficiency	
Group	Pre	Post	Pre	Post	Pre	Post	Pre	Post
Intervention (*n* = 26)	17.27 ± 4.55	29.27±5.75	39.38±11.08	41.31±10.24	2.12±0.993	4.54±1.10	58.77±13.19	72.88±13.19
Control (*n* = 24)	17 ± 5.35	16.92±5.71	39.12±10.20	38.54±10.57	1.67±1.09	1.17±0.761	57.79±11.03	56.63±11.36

Table 7 presents the outcomes of the repeated measures ANOVA, facilitating a comparison of gross motor skills, fine motor skills, upper limb coordination and general motor skills across the test stages within both the intervention and the control groups. The results of the Shapiro–Wilk test showed that the data distribution in all three variables of gross motor efficiency, fine motor efficiency and upper limb coordination was normal in both intervention and control groups and in both pre‐test and post‐test stages (0.09 < *P *< 0.321). The results of Levine's test also showed that there was homogeneity of variance between the intervention and control groups in all three aforementioned variables and in both pre‐test and post‐test stages (0.323 < *P *< 0.865).

**TABLE 7:** Outcomes of repeated measures ANOVA for comparing motor skills and their subscales in intervention and control groups across test stages.

Source	Sum of squares	df	*F*	*P*	Partial η^2^
Gross motor proficiency					
Test	886.123	1	271.061	0.001	**0.8**5
Test × Intervention	911.083	1	278.696	0.001	**0.8**53
Intervention	994.093	1	18.358	0.001	**0.2**77
Error	156.917	48			
Fine motor proficiency					
Test	11.2	1	0.682	0.413	**0.0**14
Test × Intervention	39.2	1	2.388	0.129	**0.0**47
Intervention	57.124	1	0.278	0.601	**0.00**6
Error	787.84	48			
Upper‐limb coordination					
Test	23.077	1	60.952	0.001	**0.55**9
Test × Intervention	53.317	1	140.824	0.001	**0.7**64
Intervention	91.081	1	56.332	0.001	**0.5**4
Error	18.173	48			
Total motor proficiency					
Test	1046.256	1	52.477	0.001	**0.5**22
Test × Intervention	1457.296	1	73.094	0.001	**0.6**04
Intervention	1854.031	1	6.585	0.013	**0.1**21
Error	956.994	48			

As Table 7 reveals, the interaction effect (test × group) attained statistical significance for the metrics encompassing gross motor skills, upper limb coordination and general motor skills. Consequently, to explore the disparities between the intervention and control groups across the various test stages, independent *t*‐tests were performed. Similarly, to gauge differences within each group across the test stages, paired *t*‐tests were conducted. In the pre‐test stage, no significant divergence was observed between the intervention and control groups across all four indicators (gross motor skills, fine motor skills, upper limb coordination and general motor skills). Nevertheless, significant differences emerged in the indices of gross motor skills (*t* = 7.613, *P *= 0.001, Cohen's *d* = 2.155), upper limb coordination (*t* = 12.652, *P *= 0.001, Cohen's *d* = 3.581) and general motor skills (*t* = 4.651, *P *= 0.001, Cohen's *d* = 1.317) between the intervention and control groups. Additionally, the paired *t*‐test results indicate that within the intervention group, significant differences were observed across all indices except fine motor skills between the pre‐test and post‐test stages. These encompass gross motor skills (*t* = 21.42, *P *= 0.001, Cohen's *d* = 2.125), upper limb coordination (*t* = 12.520, *P *= 0.001, Cohen's *d* = 2.301) and general motor skills (*t* = 8.885, *P *= 0.001, Cohen's *d* = 1.071). However, within the control group, a unique significant divergence occurred in the upper limb coordination index between pre‐test and post‐test (*t* = 3.391, *P *= 0.003, Cohen's *d* = 0.469).

## 4 DISCUSSION

The present study investigated the impact of an 8‐week corrective and developmental game‐based intervention on balance, coordination, sense of knee joint position and overall motor proficiency in individuals with Down syndrome. The findings revealed significant improvements in static and dynamic balance, as measured by the Y test, Romberg test and Stork test. Notably, the intervention group demonstrated superior performance compared to the control group in the post‐test phase. These results align with previous research highlighting the effectiveness of task‐oriented training in enhancing movement patterns in Down syndrome (Arvin et al., 2020; Karimian Mazidi & Nurasteh, 2023). However, there was no significant difference observed in fine motor proficiency. This discrepancy may be due to the nature of the games used, which primarily targeted gross motor skills rather than the fine motor tasks, and it may be attributed to differences in intervention protocols, duration and outcome measures used across studies. Research studies have provided evidence that adults with Down syndrome exhibit significant enhancements in balance, coordination, sense of position and overall motor proficiency following their engagement in a play‐based training programme (Syahputri & Sukoco, 2020; Valencia‐Jiménez et al., 2023).

The study also found significant enhancements in position sense under both open and closed eye conditions, two‐hand coordination, preferred hand coordination, gross motor proficiency, upper limb coordination and total motor proficiency following the intervention. These findings extend the existing literature by demonstrating the comprehensive benefits of game‐based training on various motor domains in adults with Down syndrome (Perrot et al., 2021). The results are consistent with previous studies that have reported improvements in coordination and overall motor proficiency following exercise interventions in individuals with Down syndrome (Tsimaras et al., 2012). The attractiveness of games as an exercise intervention is noteworthy, as it promotes higher compliance and motivation among individuals with Down syndrome, especially those above the age of puberty. This suggests that incorporating games into exercise protocols can encourage consistent participation and ultimately lead to improvements in the targeted components (Azab et al., 2022).

The focus on balance in the present study is justified, as imbalance resulting from inactivity can lead to a decline in muscle strength, flexibility, coordination and the sense of position. By addressing balance through corrective games, individuals can mitigate these negative effects and enhance their physical abilities (Asonitou et al., 2018). The results of this study align with other research that indicates subjects with Down syndrome experienced significant improvement in their balance after engaging in a training programme primarily focused on balance exercises (Shin et al., 2021). For instance Larkin, 2022 found that a game implemented after standard occupational therapy led to a greater improvement in dynamic standing tolerance. Additionally, another study involving community‐living adults demonstrated a significant enhancement in balance and functional mobility following a corrective exercise programme (Gheitasi et al., 2019).

Playing sports serves multiple objectives, including the development of fine and gross motor skills, enhancement of visual and auditory abilities, and improvement in attention span, coordination and movement pace. Additionally, sports also contribute to increasing body balance and, most importantly, improving autonomy (Barbu et al., 2021). Physical and corrective games are commonly recommended to facilitate the acquisition and refinement of gross motor skills, aiming to achieve independence in mobility and enable active participation in play and recreational activities (Moriello et al., 2020; Syahputri et al., 2020).

It is worth noting that while the study showed significant improvements in various physical attributes, no significant difference was found in fine motor proficiency. Fine motor proficiency is an important aspect of overall motor functioning, as it influences the ability to perform precise and coordinated movements required for daily activities (Alesi et al., 2018). Additionally, incorporating occupational therapy techniques specifically designed to enhance fine motor skills could be considered in conjunction with the game‐based intervention (Hocking et al., 2016). This finding suggests that additional interventions or targeted exercises may be necessary to specifically address fine motor skills in individuals with Down syndrome. Further research is needed to explore specific approaches that can effectively enhance fine motor proficiency in this population, ensuring a comprehensive improvement in all aspects of motor skills.

Despite these positive outcomes, the study has several limitations. The sample size was relatively small, limiting the generalizability of the findings. Additionally, the intervention duration was only 8 weeks, which may not be sufficient to observe long‐term effects. Also, the lack of a long‐term follow‐up assessment prevents determination of the sustainability of the observed improvements. Future studies should consider longer intervention periods and larger sample sizes to validate these findings further and include follow‐up assessments to evaluate the long‐term effects of the intervention.

One of the strengths of this study is the use of a game‐based intervention, which has been shown to increase engagement and motivation among participants. This approach could be applied to other populations with similar needs. Additionally, the study provides a comprehensive assessment of various motor skills and balance, contributing valuable data to the existing literature on Down syndrome and physical interventions. The inclusion of adults in the sample is another notable strength, as it expands the existing knowledge on the effectiveness of game‐based interventions across different age groups in Down syndrome.

In conclusion, this study provides evidence for the efficacy of an 8‐week corrective and developmental game‐based intervention in improving balance, coordination, position sense and overall motor proficiency in individuals with Down syndrome. The findings highlight the potential of game‐based approaches as an engaging and effective means of enhancing motor skills in this population. Future research should build upon these results by exploring strategies to target fine motor proficiency, investigating the long‐term effects of the intervention, and employing larger sample sizes with blinding to strengthen the evidence base. Nonetheless, this study contributes to the growing body of literature supporting the use of game‐based interventions for individuals with Down syndrome and underscores the importance of targeting specific motor domains to optimize their physical functioning and overall well‐being.

### 4.1 Conclusion

This study highlights the effectiveness of an 8‐week game‐based intervention in improving balance, coordination, proprioception and gross motor skills in individuals with Down syndrome. Significant gains were observed in static/dynamic balance and upper limb coordination, though fine motor skills showed no improvement. Limitations include short duration and small sample size. Future research should incorporate fine motor‐focused activities and long‐term follow‐ups. These findings support using engaging, game‐based approaches to enhance motor skills in Down syndrome therapy.

## AUTHOR CONTRIBUTIONS

Alireza Rezaee: Conceptualization, Data curation, Formal Analysis, Investigation, Methodology, Project administration, Resources, Writing—original draft, Writing—review & editing. Hasan Daneshmandi: Data curation, Formal Analysis, Investigation, Methodology, Project administration, Resources, Writing—original draft, Writing—review & editing. Hesam Ramezanzade: Data curation, Project administration, Sofware, Writing—original draft, Writing—review & editing. Sahar Mohammadzade: Data curation, Project administration, Resources, Sofware, Writing—original draft, Writing—review & editing. Mert Kurnaz: Conceptualization, Supervision, Validation, Visualization, Writing—original draft, Formal Analysis, Methodology, Writing—review & editing. Mustafa Altınkök: Conceptualization, Supervision, Formal Analysis, Methodology, Supervision, Writing—original draft, Writing—review & editing.

## CONFLICT OF INTEREST

None declared.

## FUNDING INFORMATION

No funding was received for this work.

## Supporting information



Supporting Information.

## Data Availability

The datasets used and/or analysed during the current study are available from the corresponding author on reasonable request.
